# Short‐term results of robot‐assisted colorectal cancer surgery using Senhance Digital Laparoscopy System

**DOI:** 10.1111/ases.13064

**Published:** 2022-04-28

**Authors:** Megumi Sasaki, Yasumitsu Hirano, Hiroki Yonezawa, Satoshi Shimamura, Atsuko Kataoka, Takatsugu Fujii, Naoto Okazaki, Shintaro Ishikawa, Toshimasa Ishii, Katsuya Deguchi, Hiroshi Sato, Shinichi Sakuramoto, Kojun Okamoto, Isamu Koyama

**Affiliations:** ^1^ Department of Gastroenterological Surgery Saitama Medical University International Medical Center Hidaka Japan

**Keywords:** colorectal cancer, robot‐assisted surgery, Senhance Digital Laparoscopy System

## Abstract

**Background:**

The Senhance Digital Laparoscopy System (Asensus Surgical Inc, Morrisville, NC, United States), which was introduced for the first time in Japan by our hospital, is a new surgical assistive robot following the da Vinci Surgical System. We herein report the short‐term outcomes of 55 colorectal cancer surgery cases using this system at our hospital to assess the feasibility and safety of our procedures.

**Materials and Methods:**

We retrospectively reviewed the patient backgrounds and surgical outcomes of 55 patients who underwent Senhance‐assisted laparoscopic colorectal cancer surgery.

**Results:**

The median age was 71 years. There were 31 males and 24 females, and the median body mass index was 23.1 kg/m^2^. Fifteen patients had a history of abdominal surgery. The most common surgical technique was ileocecal resection (18 cases, 32.7%), followed by high anterior resection (11 cases, 20.0%). D2 or D3 dissection was performed in each operation, and D3 dissection was performed in 41 cases (74.5%). The median operative time was 240 minutes, the median blood loss was 5 mL, there were no intraoperative complications, and there were no cases of intraoperative blood transfusion. The median postoperative hospital stay was 7 days, which was comparable to conventional laparoscopic surgery. Postoperative complications of grade 2 or higher in the Clavien–Dindo classification were observed in two cases.

**Conclusion:**

The short‐term results of 55 colorectal cancer surgery cases using the Senhance Digital Laparoscopy System were excellent and the system was introduced and surgery was safely performed.

## 1 INTRODUCTION

In recent years, the development of surgical assistive robots has been steadily progressing in the medical device market,^1^ and the Senhance Digital Laparoscopy System, which was introduced for the first time in Japan by our hospital, is a new surgical assistive robot following the da Vinci Surgical System.

The Senhance surgical robotic system (Asensus Surgical Inc, Morrisville, NC, United States) has been approved in Japan since July 2019 for insurance coverage as a laparoscopic assistance device for 98 different surgical procedures, including laparoscopic surgery for colorectal cancer. We have been using the system clinically in gynecology and urology since June 2017, mainly in colorectal cancer surgery. The recently introduced Senhance surgical robotic system can transmit haptic feedback to the surgeon, and provides an eye‐tracking camera control system, operator comfort, and reusable endoscopic instruments that enable starting the surgery with uncomplicated and highly standardized procedures. This system has already been used clinically in Europe and the United States, and there have been reports of its use in many surgical procedures, including colorectal cancer surgery.^2^, ^3^, ^4^, ^5^, ^6^ However, it has only been introduced at four institutions in Japan, including our hospital. We herein report the short‐term outcomes of 55 colorectal cancer surgery cases using this system at our hospital to assess the feasibility and safety of our procedures.

## 2 MATERIALS AND METHODS

This study was approved by the clinical research institutional review board of Saitama Medical University International Medical Center (approval no. 2021‐088) and was performed in accordance with the 1964 Declaration of Helsinki and its later amendments or specified comparable standards.

We retrospectively reviewed the backgrounds and surgical outcomes of 55 patients who underwent Senhance‐assisted laparoscopic colorectal cancer surgery at our department between May 2018 and May 2021.

### 2.1 Surgical technique

**FIGURE 1:**
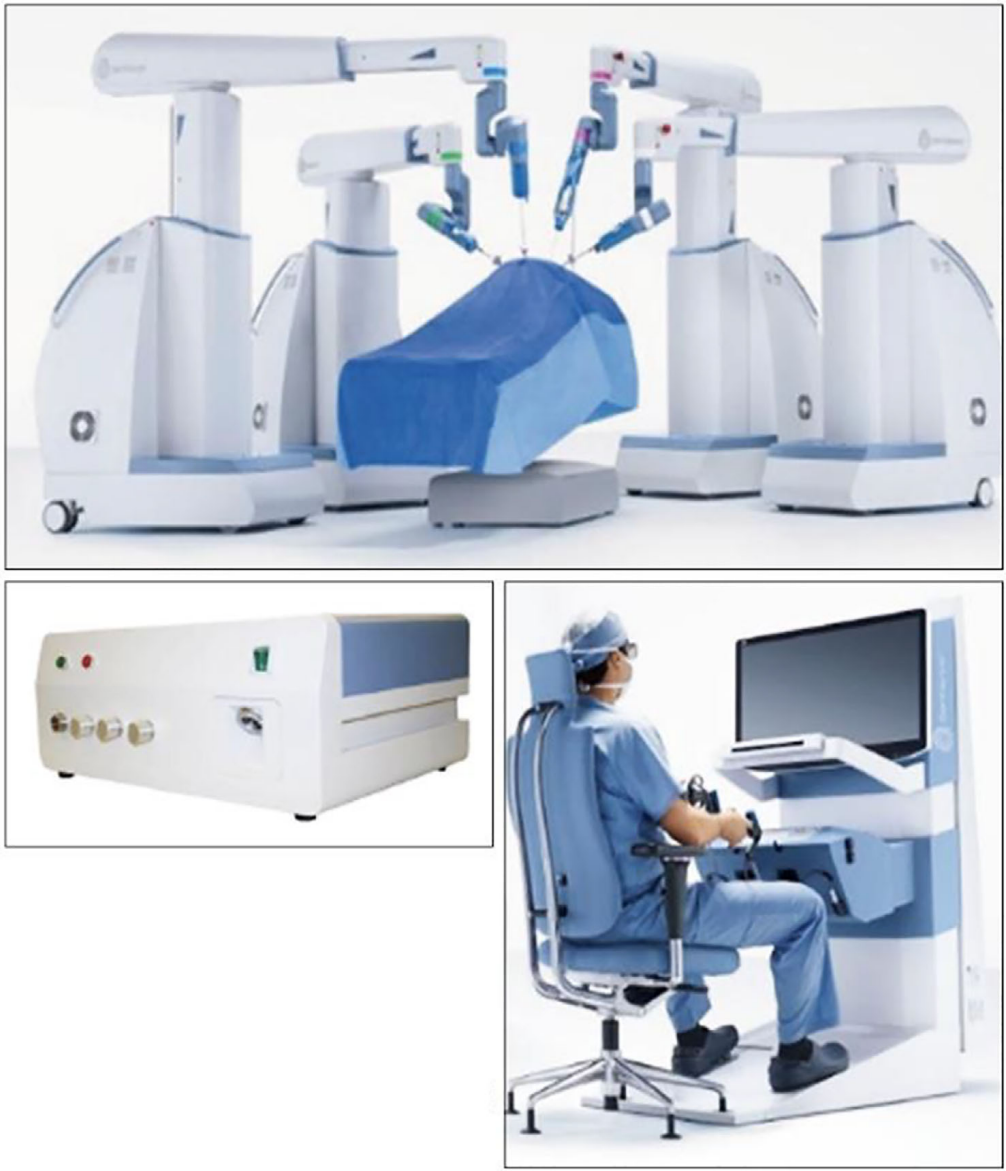
Senhance Digital Laparoscopy System

**FIGURE 2:**
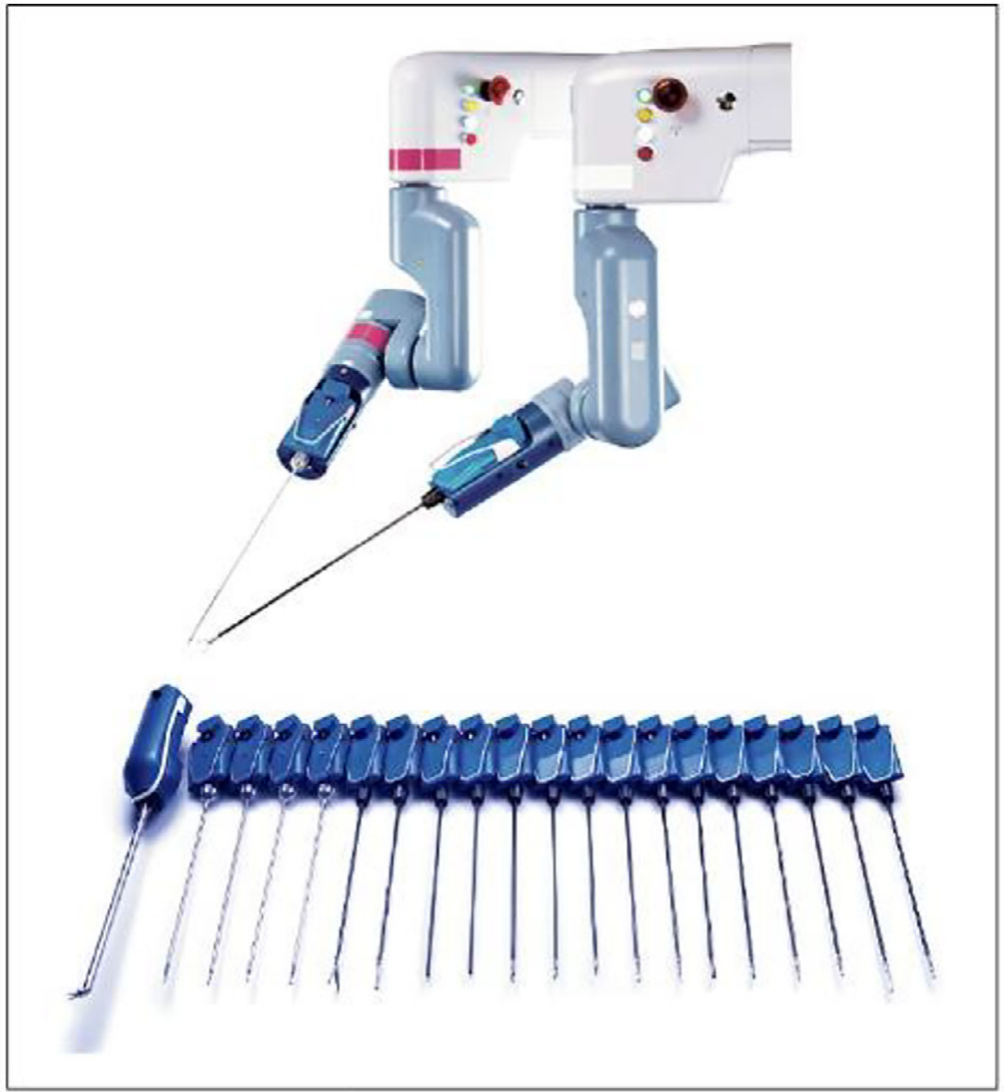
The forceps of Senhance Digital Laparoscopy System

The Senhance system consists of a stand‐alone manipulator arm, a cockpit, and a computer node that controls digital signals from the manipulator arm and cockpit and video signals from the endoscopic camera system. (Figure 1) The forceps can be attached to any arm, and various areas can be approached by appropriately changing the arm to which the scope and forceps are attached (Figure 2).

### 2.2 Type A (15 cases: conventional method with assistance by assistant forceps)

**FIGURE 3:**
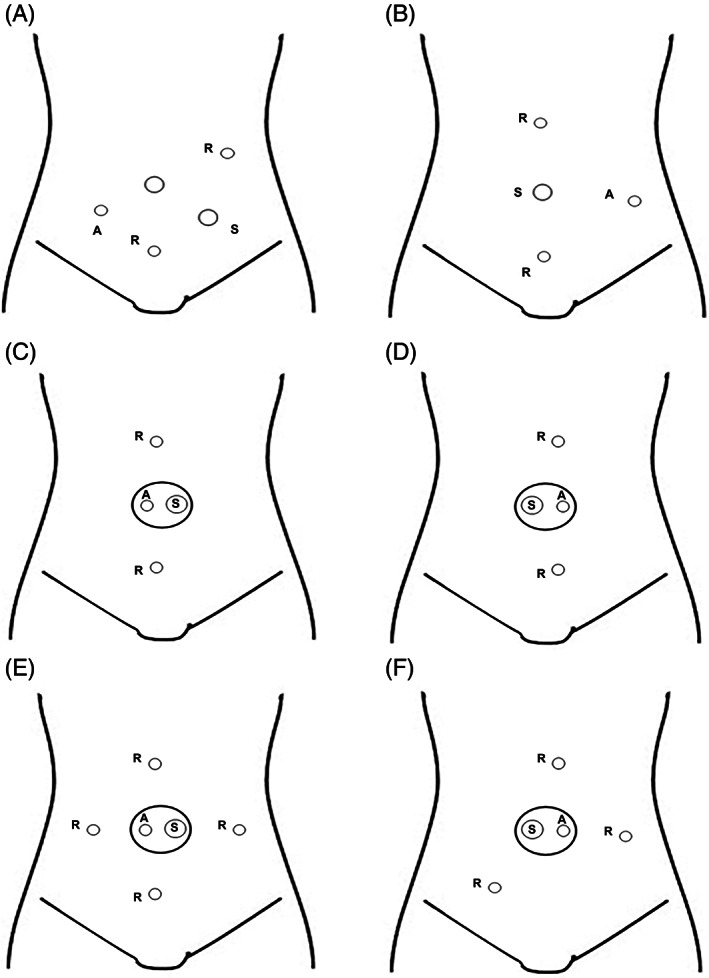
Port placement. (A) Right side colon, (B) left side colon, (C) ileocecal resection, (D) sigmoid colectomy, (E) right hemicolectomy, (F) lower anterior resection. R: 5‐mm port robotic forceps, S: 12‐mm camera port, A: 5‐mm port assistant forceps

Ports were constructed based on conventional port placement in laparoscopic colorectal cancer surgery in our department. The port arrangement using this system is shown in Figure 3A, B.

### 2.3 Type B (40 cases: reduced‐port surgery method without assistance)

A 2.5 cm longitudinal incision is made at the umbilicus and a small laparotomy is performed. A 12‐mm port is inserted with Free Access® and a 5‐mm port for the surgeon's forceps is inserted as shown in Figure 3C–F. The surgical technique followed the method of reduced‐port surgery, and the procedure was essentially completed with Senhance forceps alone without an assistant. A 5‐mm port was added to the Free Access to assist in intraoperative maneuvers when assistance was needed. Regardless of the completion of the procedure with this system, we did not hesitate to shift to laparoscopic surgery for planned partial use or in the case of difficulties.

In surgery for cecal cancer, some ascending colon cancers, sigmoid colon cancer, and rectal sigmoid cancer, the technique consists of lymph node dissection of the ileocolic artery or inferior mesenteric artery and transfer of the intestine, including the mesentery. In these cases, the operation is performed through a small incision at the umbilicus and a free access port placed in a straight line above and below the incision (Figure 3C, D). In right hemicolectomy, the dissection around the middle colonic artery is performed from caudad to cephalad, and in rectal cancer, the dissection around the rectum is performed from cephalad to caudad. Therefore, we performed the procedure through a small incision in the umbilicus and placed three ports as shown in Figure 3E, F.

## 3 RESULTS

Patient background is shown in Table 1. The median age was 71 years, 31 patients were male and 24 were female. The median body mass index was 23.1 kg/m^2^. Fifteen patients had a history of abdominal surgery. The type of operation performed is shown in Table 2 and surgical results are shown in Table 3. The most common surgical technique was ileocecal resection (18 cases, 32.7%), followed by high anterior resection (11 cases, 20.0%). D2 or D3 dissection was performed in each operation, and D3 dissection was performed in 41 cases (74.5%). The median number of lymph nodes removed was 21. The median operative time was 240 minutes, the median blood loss was 5 mL, there were no intraoperative complications, and there were no cases of intraoperative blood transfusion. The median postoperative hospital stay was 7 days, which was comparable to conventional laparoscopic surgery. Postoperative complications of grade 2 or higher in the Clavien–Dindo classification were observed in two cases: one case of perianastomotic blood infection and one case of suture failure. Both patients had comorbidities including diabetes mellitus, but both were cured by conservative treatment.

**TABLE 1:** Characteristics of the study patients

	55 cases
Median age, y (range)	71 (39–91)
Gender, male/female	31/24
Median body mass index (range)	23.1 (14.0–34.2)
History of abdominal surgery, +/−	15/40
Operation type, A/B	15/40
ASA, I/II/III	15/36/4
Tumor location, V/C/A/T/S/R	2/11/12/2/15/13
cT stage, cTis/1/2/3/4	2/8/10/30/5
cN stage, cN0/1/2/3	40/11/2/2
cStage, 0/I/II/III	2/17/21/15

Abbreviation: ASA, American Society of Anesthesiologists classification.

**TABLE 2:** Types of operation performed

Operation	55 cases (%)
Ileocecal resection	18 (32.7)
Right hemicolectomy	8 (14.5)
Transverse colectomy	1 (1.8)
Sigmoid colectomy	9 (16.3)
High anterior resection	11 (20.0)
Low anterior resection	7 (12.7)
Intersphincteric resection	1 (1.8)

**TABLE 3:** Surgical results

	55 cases
Operation time, min (range)	240 (101–378)
Docking time, min (range)	8 (2–25)
Senhance operating time, min (range)	126 (24–287)
Blood loss, mL (range)	5 (0–167)
Lymph node dissection, D2/D3	14/41
Number of dissected lymph nodes median (range)	21 (7–39)
fStage, 0/I/II/III/IV	3/17/18/15/2
Postoperative stay, d (range)	7 (5–27)

## 4 DISCUSSION

In robot‐assisted surgery with Senhance, more delicate and precise surgical procedures can be performed using the hands movement removal and motion scaling functions. Moreover, the quality of surgical procedures is expected to improve because surgeons can perform surgery and simultaneously view stable high‐resolution 3D images. Senhance, a surgical support robot developed after the da Vinci system, is a system that digitizes the interface between the surgeon and patient in a conventional laparoscopic surgery. Table 4 shows a comparison between the Senhance and da Vinci systems. The development goal of Senhance is to digitize the current laparoscopic surgery, and da Vinci aims to perform more accurate surgery. Therefore, they are very different methods. In Senhance, in contrast to da Vinci, many forceps can be reused without any restrictions on the number of times they are used, and the port can be used as it is for laparoscopic surgery, which is used daily at each facility. Cost reduction can be expected when using Senhance. This usage cost is not substantially different from that of a normal laparoscopic surgery, and thus, Senhance can be practically used for procedures, such as “lymph node dissection only” and “mobilization only,” at the initial stage of introduction, thereby enabling a reasonable and safe introduction. Because the forceps operation with Senhance can be performed with almost the same approach as conventional laparoscopic surgery, there is no need to learn new forceps operation when introducing Senhance. Furthermore, we believe that the tactile feedback system may prevent inadvertent organ damage, and da Vinci does not offer this function. Other features include camera control with the movement of the operator's eyes, reduction of the burden on the operator's neck and back as the surgical operation posture is not fixed, and the shape of the tip of the forceps which is the same as the forceps for conventional laparoscopic surgery. However, the shape of the tip of these forceps is the same as that used for a laparoscopic surgery, and thus, it is difficult to perform a peeling operation with smooth movements such as those using human fingers and wrists in da Vinci. This is one of the major drawbacks of Senhance. Therefore, it seems that da Vinci surgery is suitable for cases requiring suture ligation in the deep pelvis and for particularly difficult procedures requiring dissection at the pelvic floor, such as lower rectal cancer. Moreover, because the range of motion of Senhance's robot arm is limited, it may be difficult to complete the procedure depending on the location of the lesion and the physique of the patient. However, the use of forceps that can bend the tip, which has already been applied overseas, is expected to improve surgical techniques and be increasingly used for colorectal cancer surgery. Furthermore, with the additional introduction of intelligent surgical unit (ISU), which is a function utilizing artificial intelligence, the computer interprets the digitized visual information of the surgical field captured in the platform and recognizes the position of the field and object. The ISU recognizes the tip of the forceps, controls the camera to ensure that the tip is at the center of the field‐of‐view (Follow Me) without the need for instructions from the operator, and recognizes the position indicated by the operator with the forceps. It is equipped with the ability to move the camera (To Go), which is expected to be the first step in introducing extended intelligence and machine vision into robotic surgery.

**TABLE 4:** System characteristics

Device	da Vinci	Senhance
Console	Closed type	Open type
Optics	8 mm 3D	10 mm 3D
Forceps size	8 mm	3 mm or 5 mm
Haptic feedback	No	Yes
Field‐of‐view control	Handle + foot pedal	Handle button + eye movement
Reuse	Up to 10 times	Possibly without limit

In Europe and the United States, this system has already been used in numerous surgeries, such as in urology and gynecology, and some reports have indicated that it can be safely used in the gastrointestinal tract. Spinelli et al^6^ and Samalavicius et al^7^ have reported from Europe on surgery using this system for colorectal cancer; however, some reports indicate that lymph node dissection was performed in vitro because of a small number of cancer cases. In Asia, Lin et al^8^ from Taiwan recently reported 46 cases of colorectal resection, and the short‐term results of colorectal cancer, which accounted for 39 cases, showed attention to indications and patient selection. In the short‐term results of 55 patients who underwent colorectal cancer surgery by using this device in our department, no problems were observed with regards to bleeding volume, dissection range, number of dissected lymph nodes, etc. In addition, the length of hospital stay after surgery and the incidence of complications were similar to that of a conventional laparoscopic surgery. The number of cases in this study is limited to 55, and we believe that further cases will need to be accumulated in the future. Although the 55 cases in this study include the partial use of dissection alone, the number of cases will be increased in the future. We will further consider the evaluation of the increased learning curve.

Compared with da Vinci using articulated forceps, Senhance has less merit in high‐difficulty surgery, such as rectal cancer surgery, and its introduction to Japan is still in the testing stage. Because reproducing the surgical technique established by the conventional method is easy, it can be used for performing relatively low‐difficulty surgeries more safely and comfortably in a facility with few doctors. Currently, Senhance is introduced only in seven facilities in Japan. Senhance aims to digitize and improve laparoscopic surgery, and this digital laparoscopy system is different from other surgery support robots, including da Vinci. Because it is a system in a new area, the corporate strategy of the distributor Asensus is to limit the facilities and collect cases safely at this time. Therefore, it has not been officially released in Japan yet, and it is being introduced in a limited number of facilities.

Further improvements to this system, such as functional complementation by the evolution of ISU in the future and the introduction and effective utilization of forceps with bending tips, are expected to establish more useful minimally invasive surgery with ergonomic consideration for surgeons.

## CONFLICT OF INTEREST

The authors declare no conflicts of interest.

## AUTHOR CONTRIBUTIONS

MS and YH wrote the manuscript. IK supervised this report. All authors reviewed the manuscript.

## FUNDING INFORMATION

The authors did not receive any funding for this study.

## Data Availability

Research data are not shared.
